# A genetically determined molecular switch modulates the anti-inflammatory potential of human IgA

**DOI:** 10.3389/fimmu.2025.1641351

**Published:** 2025-08-27

**Authors:** Andrew W. Gibson, Jianming Wu, R. Curtis Hendrickson, Travis Ptacek, James Mobley, Jeffrey C. Edberg, Robert P. Kimberly

**Affiliations:** ^1^ Division of Clinical Immunology and Rheumatology, Department of Medicine, The University of Alabama at Birmingham, Birmingham, AL, United States; ^2^ Department of Microbiology, The University of Alabama at Birmingham, Birmingham, AL, United States; ^3^ Department of Anesthesiology and Perioperative Medicine, The University of Alabama at Birmingham, Birmingham, AL, United States

**Keywords:** FCAR, alleles, signal transduction, CD89, inhibition, SH3BP5 (Sab)

## Abstract

Fc receptor-driven immune system activity typically reflects a balance of activating and inhibitory mechanisms, mediated by the immunoreceptor tyrosine-based activation motif (ITAM) or inhibition motif (ITIM) in the ligand-binding alpha chain (FcγRIIa – c) or the canonical ITAM in the associated Fc receptor γ-chain (FcRγ). A second role for the ITAM, an inhibitory role known as ITAM_i_, was initially recognized for the FcαRI-FcRγ signaling pair. We report an FcRγ-independent mechanism for inhibitory signaling by the IgA-binding receptor, FcαRI (CD89) in which the natural Ser^248^Gly variant in the cytoplasmic domain of the FcαRI α-chain alters the signaling capacity of FcαRI and constitutes a serine-based genetically determined switch for regulation of the anti- and proinflammatory potentials of human IgA. To elucidate the basis for this α-chain mechanism, we sought allele-specific FcαRI-associated molecules. Sab (SH3BP5), a trans-inhibitor for Bruton’s tyrosine kinase (Btk), is recruited by the more common Ser^248^ allele, whereas the src-family tyrosine kinase Lyn, a Btk activator, is reciprocally recruited by the Gly^248^ variant. Ser^248^ phosphorylation amplifies Sab association and disrupts Lyn binding through an overlapping region containing an unconventional SH3-domain binding motif. In contrast to FcαRI Gly^248^, recruitment of Sab by FcαRI Ser^248^ results in inhibition of Btk activation and suppression of IgA effector functions independent of FcRγ-pairing. Expression of a dominant-negative Sab construct releases FcαRI-mediated inhibition in a Ser^248^- allele-specific manner. These findings reveal a reversible serine-based phosphorylation-dependent molecular switch for regulation of receptor-mediated activation/inhibition that couples FcαRI α-chain to divergent inflammatory properties of human IgA.

## Introduction

The FcαRI protein (CD89) binds to its cognate ligands, IgA and C-reactive protein (CRP), and can interact directly with both gram-positive and gram-negative bacterial species. This binding mediates effector functions (1, 2). FcαRI is expressed constitutively on myeloid cells, including neutrophils, monocytes, macrophages, eosinophils, and a subpopulation of dendritic cells (1, 3–5). The receptor is also expressed on Kupffer cells, where it is thought to play a role in IgA nephropathy (6). Structurally, FcαRI comprises two extracellular Ig-like domains, a transmembrane region, and a cytoplasmic tail devoid of any classically recognized signaling motifs. However, the receptor can associate noncovalently with and signal through the FcRγ chain (1, 7, 8), which contains a canonical immunoreceptor tyrosine-based activation motif (ITAM) that recruits kinases and phosphatases and initiates a signaling cascade that culminates in downstream cellular effector functions. These effector functions include phagocytosis, antigen presentation, respiratory burst, and the release of proinflammatory mediators and cytokines (9–11).

Paradoxically, reports also indicate that serum IgA, through FcαRI-binding, may mediate anti-inflammatory functions, including downregulation of phagocytosis, chemotaxis, bacterial killing, cytokine release, oxidative bursts, and inhibition of lipopolysaccharide (LPS)-, IgG-, C5a-, and formyl-methionine-leucine-phenylalanine (fMLP)-induced cytokine release (12–17). The discovery of an inhibitory ITAM function (ITAM_i_) as a mechanism for negatively controlling immune responses (18–20) might explain some of these empirical observations of anti-inflammatory function. The ITAM of the FcαRI-associated FcRγ chain can mediate inhibition depending on the degree of receptor oligomerization, with low-order oligomers (e.g., dimerization) of FcαRI-FcRγ delivering inhibitory signals (20, 21). However, unlike the FcRγ-associated IgG Fc receptors, FcγRI and FcγRIII, FcαRI does not require pairing with FcRγ for expression (22) and is typically expressed as a single ligand-binding α-chain on resting neutrophils (23, 24). Whether the α-chain cytoplasmic domain of FcαRI plays a role in receptor signaling mode was not investigated in these studies and remains unclear.

The *FCAR* gene encodes the FcαRI (CD89) protein. A single nucleotide polymorphism at nucleotide position (rs16986050; nt 844A>G) in the *FCAR* gene changes amino acid residue 248 in the receptor protein’s cytoplasmic domain from serine to glycine and significantly alters receptor function. The FcαRI Gly^248^ α-chain, expressed in the absence of FcRγ-chain pairing, has a highly activating signaling capacity (25, 26). In contrast, the FcαRI Ser^248^ allele, also in the absence of FcRγ-chain pairing, lacks this activating potential and mediates inhibition. While direct recruitment of Lyn kinase to the FcαRI Gly^248^ α-chain may explain activation signals for that allelic form (25), the basis for the FcαRI Ser^248^ allele inhibition remains undefined.

To explore the molecular basis of differential signaling by the FcαRI-Ser^248^Gly α-chains, we sought S^248^G allele-specific FcαRI-binding molecules. Our results show that FcαRI Ser^248^ downregulates many effector functions in the absence of FcRγ-chain pairing, while FcαRI Gly^248^ activates downstream functions independent of FcRγ chain. Our results show that Sab (SH3 homology associated Btk binding protein 5 [SH3BP5]), a trans-inhibitor of Bruton’s tyrosine kinase (Btk), is exclusively recruited by FcαRI Ser^248^ and inhibits downstream functions. Furthermore, we show that phosphorylation of Ser^248^ by CK1δ facilitates Sab recruitment, but not Lyn binding, and that Sab and Lyn bind reciprocally to the region within the FcαRI cytoplasmic domain containing Ser^248^ and resembling an unconventional SH3-domain binding motif. This serine-based, phosphorylation-dependent mechanism of differential recruitment of signaling molecules provides a genetically dependent molecular switch that contributes to the divergent pro- and anti-inflammatory capacities of FcαRI. This mechanism may contribute to the risk for some autoimmune diseases (27) and could inform a precision medicine approach to antibody-based therapeutics, when an IgA heavy chain backbone is used (28–33).

## Methods

### Donors

Peripheral blood for *ex vivo* studies was obtained from healthy, normal volunteers. The human studies were reviewed and approved by the Institutional Review Board, and all donors provided written informed consent.

### Antibody reagents

Anti-FcαRI mAb A59 was a gift from Dr. H. Kubagawa. The anti-FcαRI mAb MIP8a was from Bio-Rad Laboratories (Hercules, CA, USA). The goat anti-Sab Ab E-18, rabbit anti-Lyn Ab, rabbit anti-Syk N-19, and antiphosphotyrosine mAb PY20 were from Santa Cruz Biotech (Santa Cruz, CA, USA). Rabbit anti-Btk M-138 and rabbit anti-phosphoTyr223-Btk were from Abcam (Eugene, OR, USA), while rabbit anti-FcRγ and the antiphosphotyrosine mAb 4G10 were from Upstate Biotech (Syracuse, NY, USA). The anti-FcγRIIa mAb 11B6, anti-FcγRIIb mAbs 3B6 and 4F5, and the anti-Sab mAb 2B5.1 were made by the Hybridoma Core Facility at the University of Alabama at Birmingham (UAB). Hybridomas secreting anti-FcγRIa mAb 32.2 and anti-FcγRIIa mAb IV.3 were from ATCC (Manassas, VA, USA), and the secreted antibodies were purified by Protein A affinity chromatography. The anti-His6G, anti-GST, and anti-CK1δ mAbs were from Thermo Fisher Scientific (Grand Island, NY, USA). The rabbit antihuman IgA, rabbit antihuman IgG, mIgG1 F(ab′)_2_, and the HRP- and fluorescein isothicyanate (FITC)-labeled secondary antibodies were from Jackson ImmunoResearch (Westgrove, PA, USA).

### Cell culture

Human peripheral blood monocytes and neutrophils were isolated from ethylenediaminetetraacetic acid (EDTA)-anticoagulated blood through two-step Ficoll–Hypaque density gradient centrifugation. Human and murine cell lines from ATCC and primary human leukocytes were cultured at 37°C with 5% CO_2_ in RPMI-1640 or DMEM (P388D1), supplemented with 10% heat-inactivated fetal bovine serum (Thermofisher, Grand Island, NY, USA). Stably transfected P388D1 or U937 cell lines were maintained in medium supplemented with 1 mg/mL of G418. Stimulation of cells was performed at a density of 1.0 × 10^6^ cells/mL or as indicated.

### Measurement of receptor expression

FcαRI surface expression was determined using F(ab′)_2_ fragments of A59. Briefly, 0.05 mL of 5 × 10^6^ cells/mL were opsonized with 20- μg/mL F(ab′)_2_ A59 for 30 min on ice, washed, and stained with 20- μg/mL FITC-labeled F(ab′)_2_ goat antimouse IgG (Jackson ImmunoResearch) on ice for 30 min. Stained cells were analyzed immediately by flow cytometry. Alternatively, we stained FcαRI using FITC-labeled MIP8a.

### Purification of human serum IgA forms

Serum IgA forms were purified from plasma pools of healthy donors using Jacalin column affinity purification (Thermo Fisher), followed by Sephacryl S-300 (Cytiva, Marlsborough, MA, USA) size -exclusion separation after a QAE Sephadex A-50 (Cytiva) ion exchange. Heat-aggregated IgA was obtained by heat treatment of human serum IgA (MilliporeSigma, St. Louis, MO, USA) at 63°C for 150 min, followed by Sephacryl S- 300 size- exclusion separation to remove unaggregated forms.

### Stimulation of FcαRI

For stimulation of FcαRI, cells were treated either with monomeric or heat-aggregated serum IgA alone at 10 to 200 µg/mL or together with LPS (Sigma, St. Louis, MO, USA). In a second paradigm, cells were cultured in tissue culture plates precoated with human serum IgA or anti-FcαRI mAb F(ab′)_2_ A59 alone (20 µg/mL or as indicated) or together with LPS as indicated. Precoating was done in 0.1- M carbonate buffer, pH 9.5, at room temperature for 6 h. In a solution, cells were opsonized with F(ab′)_2_ A59 (20 µg/mL) on ice for 30 min, followed by cross-linking at 37°C using F(ab′)_2_ goat antimouse IgG (20 µg/mL) (Jackson ImmunoResearch) in Hanks Balanced Salt Solution (HBSS) (Thermofisher, Grand Island, NY, USA) containing 5- mM glucose, 0.1% Bovine Serum Albumin (BSA), 1.08- mM CaCl_2_, 1.62- mM MgCl_2_, and 20- mM 4-(2-hydroxyethyl)-1- piperazine ethanesulfonic acid (HEPES). For monomeric or dimeric engagement of FcαRI, cells were cultured in the presence of IgA at a concentration of 10 to 200 µg/mL, or as indicated. Alternatively, cells were cultured in the presence of anti-FcαRI mAb F(ab′)_2_ A59 at a concentration of 10 µg/mL, or as indicated.

### Genotyping, cloning, and transfection

Genomic DNA isolation, PCR, RT-PCR, DNA sequencing, and single-nucleotide polymorphism (SNP) allele genotyping were performed as previously described (25). FcαRI expression constructs, GST-FcαRI cytoplasmic domain fusion protein expression constructs, and P388D1 transfectants stably expressing FcαRI were generated as described (25). The human Sab expression construct was generated by cloning *Kpn*I*/Eco*RI-flanked RT-PCR products containing 1.326 kb of the Sab coding region into pcDNA3.1 (Life Technologies). The dominant negative Sab construct (pcDNA3.1/SabΔ31) was generated from full-length pcDNA3.1/Sab using site-directed mutagenesis to truncate 31 codons of the Btk-binding site according to (34). Transient co-transfection of COS7 cells with pcDNA3/FcαRI and pcDNA3.1/Sab was conducted using the Lipofectamine 2000 reagent following the manufacturer’s suggestions (Thermo Fisher). Stable U937 cell lines expressing dominant negative Sab and control cells were established by transfection with pcDNA3.1/SabΔ31 or pcDNA3.1 vector alone, respectively, followed by selection with G418.

### Isolation and identification of FcαRI-associated molecules

FcαRI-associated molecules were isolated through co-purification with FcαRI-specific mAb affinity columns. Briefly, F(ab′)_2_ fragments of mAb A59 were coupled to Sepharose beads using the AminoLink Kit (Thermofisher). A total of 10^8^ cells were lysed on ice for 15 min using 0.5% NP-40 (Sigma) and 0.5% Triton X-100 (Sigma) in 5 mL of PBS containing protease inhibitor cocktail (MilliporeSigma) before centrifugation (20,000 × *g*, 10 min, 4°C). After preclearing with F(ab′)_2_ mIgG column, FcαRI and associated molecules were captured on the A59 column. Bound proteins were eluted sequentially with 0.1% digitonin, pH 7. 2, in PBS containing 1- M NaCl, followed by 0.1% digitonin, pH 2.8, 0.1- M glycine buffer (neutralized with 1M Tris.Cl, pH8.0), and finally with Tris· Cl, pH 6. 8, containing 0.1% sodium dodecyl sulfate (SDS) and 100- mM dithiothreitol (DTT), then concentrated by precipitation with 60% methanol. Precipitated proteins were separated by SDS-PAGE and were then visualized by silver staining or colloidal blue staining (Thermo Fisher). Protein bands of interest were excised from colloidal blue-stained gels and identified by mass spectrometry (MALDI-MS; **Supplementary Figure 1**). Proteomic analysis was performed by the Proteomics Core Facility at UAB (35).

### Immunoprecipitation and immunoblotting

Immunoprecipitation of FcαRI was performed using F(ab′)_2_ fragments of A59 conjugated to Sepharose beads. Specificity of immunoprecipitation (IP) was established using control F(ab′)_2_ fragments of mIgG conjugated to Sepharose beads. Alternatively, for selective precipitation of FcαRI expressed on the cell surface or in intracellular pools (36), we first saturated surface FcαRI using F(ab′)_2_ A59, and then precipitated surface-opsonized receptor or intracellular unopsonized receptor using F(ab′)_2_ goat anti mouse IgG-conjugated beads or F(ab′)_2_ A59 beads. For immunoblotting (IB), immunoprecipitated proteins were denatured at 95 °C for 5 min, or at room temperature overnight (phosphoTyr223-Btk), separated on SDS-PAGE, and transferred to a 0.22-μm nitrocellulose membrane (Bio-Rad Laboratories). Blots were blocked with 5% nonfat dry milk (common blot), or 3% BSA (antiphosphotyrosine 4G10), or 5% milk plus 2% BSA (antiphosphoTyr223-Btk) before probing with antibodies and detection with chemiluminescence (Pierce Biotechnology). Tyrosine phosphorylation of Btk or Syk was detected by blotting of Btk (rabbit anti-Btk M-138) or Syk (rabbit anti-Syk N-19) in antiphosphotyrosine precipitates using PY20-beads, or by immunoblotting anti-Btk or anti-Syk precipitates with antiphosphotyrosine 4G10.

### Physical association of Sab with FcαRI

Physical association of Sab with FcαRI was examined by probing for Sab in FcαRI immunoprecipitates from primary cells and cell lines expressing these proteins endogenously or through stable transfection. A total of 10^8^ COS7 co-expressing FcαRI and His-tagged Sab were lysed, and lysates were passed over a 1-mL Ni–NTA affinity column to bind His-tagged Sab. The column was washed with 20- mL lysis buffer containing 20- mM imidazole. Bound His6-Sab was then eluted with lysis buffer containing 250- mM imidazole. Proteins in the lysate, flow- through, and eluted fractions were subjected to SDS-PAGE and immunoblotted for FcαRI in each fraction.

### *In vitro* kinase assays

Phosphorylation of FcαRI cytoplasmic tail (CYT) by CK1 was performed in *in vitro* kinase assays (IVK) using ^32^P-adenosine triphosphate (^32^P-ATP), purified GST-FcαRI CYT fusion proteins, and purified CK1δ. Briefly, we initially purified His-tagged intact human CK1δ protein from *Escherichia coli* using an Ni–NTA (QIAGEN, Germantown, MD, USA) affinity column, then the protein was further purified using a hydroxyapatite gel (HT; Bio-Rad) column eluted with a 10- to 400-mM gradient phosphate buffer, pH 6.8, plus 500- mM NaCl. Purified CK1δ was activated by dephosphorylation in phosphatase buffer (50- mM Tris·Cl, pH 7.5, 10- mM MnCl_2_, 0.1- mM EDTA, and 1- mM DTT) at 30°C for 30 min using 0.5 unit of protein serine/threonine phosphatase 1 (PP1; catalytic subunit, Promega, Madison, WI, USA)/μg of CK1δ in a 50-μL reaction. To phosphorylate the GST-FcαRI CYT, 10 µg of the fusion protein was mixed with 0.05- μg PP1-treated CK1δ in 50 µL of buffer (50- mM Tris·Cl, pH 7.5, 10- mM MgCl_2_, 1- mM EDTA, 0.4- mM EGTA, and 1- mM DTT), 5 µCi of ^32^P-ATP, and 0.1 µM of microcystin-LR to inhibit PP1, and incubated at 30°C for 30 min. Following SDS-PAGE separation, phosphorylated proteins were detected by autoradiography, and nonphosphorylated protein loading equivalents were visualized by Coomassie blue staining. To phosphorylate GST-CYT for examining Sab interaction, 0.1- mM cold ATP was used in place of ^32^P-ATP in IVK reactions. Following phosphorylation, 10 µg of GST-CYT fusion proteins were adsorbed to 10 µL of glutathione Sepharose-4B, washed with CK1 buffer, and incubated with 10 µg of purified His-tagged Sab at 30°C for 60 min. Sab interaction with GST-CYT was assessed by SDS-PAGE and anti-Sab immunoblotting.

### Phagocytosis

FcαRI-mediated phagocytosis was performed using F(ab′)_2_ A59-opsonized FITC-labeled ox red blood cells (RBC). Briefly, 10^9^/mL of biotin-labeled RBC (EB) were incubated with 0.25 mg/mL of FITC-labeled streptavidin at room temperature for 30 min, and then incubated with biotin-labeled F(ab′)_2_ A59 (0.1 mg/mL) for 30 min at room temperature to create EBAB-A59. Phagocytosis was performed at 37°C by mixing 10^5^ P388D1 or U937 cells with 2 × 10^6^ EBAB-A59 in 0.2 mL of HBSS containing 1.08- mM CaCl_2_, 1.62- mM MgCl_2_, 0.1% BSA, and 10- mM HEPES. The assay was stopped at various time points by incubating on ice. Phagocytosis of FITC-labeled RBC was measured by flow cytometry (37, 38). Extracellular FITC fluorescence was quenched using Trypan blue (100 μg/mL).

### Cytokine measurements

The concentrations of cytokines [interleukin (IL)-6, tumor necrosis factor alpha (TNF-α), or IL-1β] secreted in the culture supernatants were measured by ELISA using the cytokine detection ELISA kits according to the manufacturer’s instructions (BD Biosciences, Franklin Lakes, NJ, USA).

### Genomic and bioinformatics analyses

Whole exome sequencing was performed on 983,578 donors, and variants were called and filtered as described (39). Variant call files (VCF) were downloaded for chromosomes 1 and 19 (release 20231004), and variant impact was annotated with snpEff 5.0e using dbSNP 156 (40) and a version of Ensembl (release 111) (41) was modified to annotate FCGR2C (ENST00000466542.6) as a coding gene rather than a pseudogene. Variants were then filtered to include only those causing nonsynonymous coding changes to the cytoplasmic domain (**Supplementary Figure 2**), as annotated in the canonical transcript for each gene (42) (https://github.com/KimberlyLab/cd89paper_rgc_me_variant_analysis).

### PacBio circular consensus sequencing and analyses

Fastq files from the Arab pangenome project and assembled genomes from the Chinese pangenome project were obtained through the Sequence Read Archive (SRA) with facilitation by the projects’ respective directors. Fastq files for SRA donors were downloaded from the NCBI website. In addition, selected samples from the CASSLE cohort of SLE persons and normal controls, and from the CSER study, were sequenced at the Hudson Alpha Institute for Biotechnology (43, 44). Data from 215 donors were aligned to the hg38 reference genome using Minimap2 2.28-r1209 (45). Samtools 1.21 was used to extract reads overlapping the low-affinity *FCGR* gene cluster (chr1:161,484,000–161,789,000) from the Binary Alignment Map (BAM) file resulting from the initial alignment. These reads were passed to Hifiasm 0.20.0-r639 for *de novo* assembly (46–48). Assemblies passed QC for 201 donors (**Supplementary Table 1**). The entire workflow is implemented in Snakemake pipelines (49).

### Statistical analysis

Statistical significance was assessed by Student’s *t*-test, paired or unpaired as indicated. Differences with *p*-values less than 0.05 were considered significant at the ^*^ *p* < 0.05 or ^**^ *p* < 0.01 and *** *p* < 0.001 levels.

## Results

### FcαRI Ser^248^/Gly^248^ alleles alter receptor capacity to signal proinflammatory cytokine production

**Figure 1:**
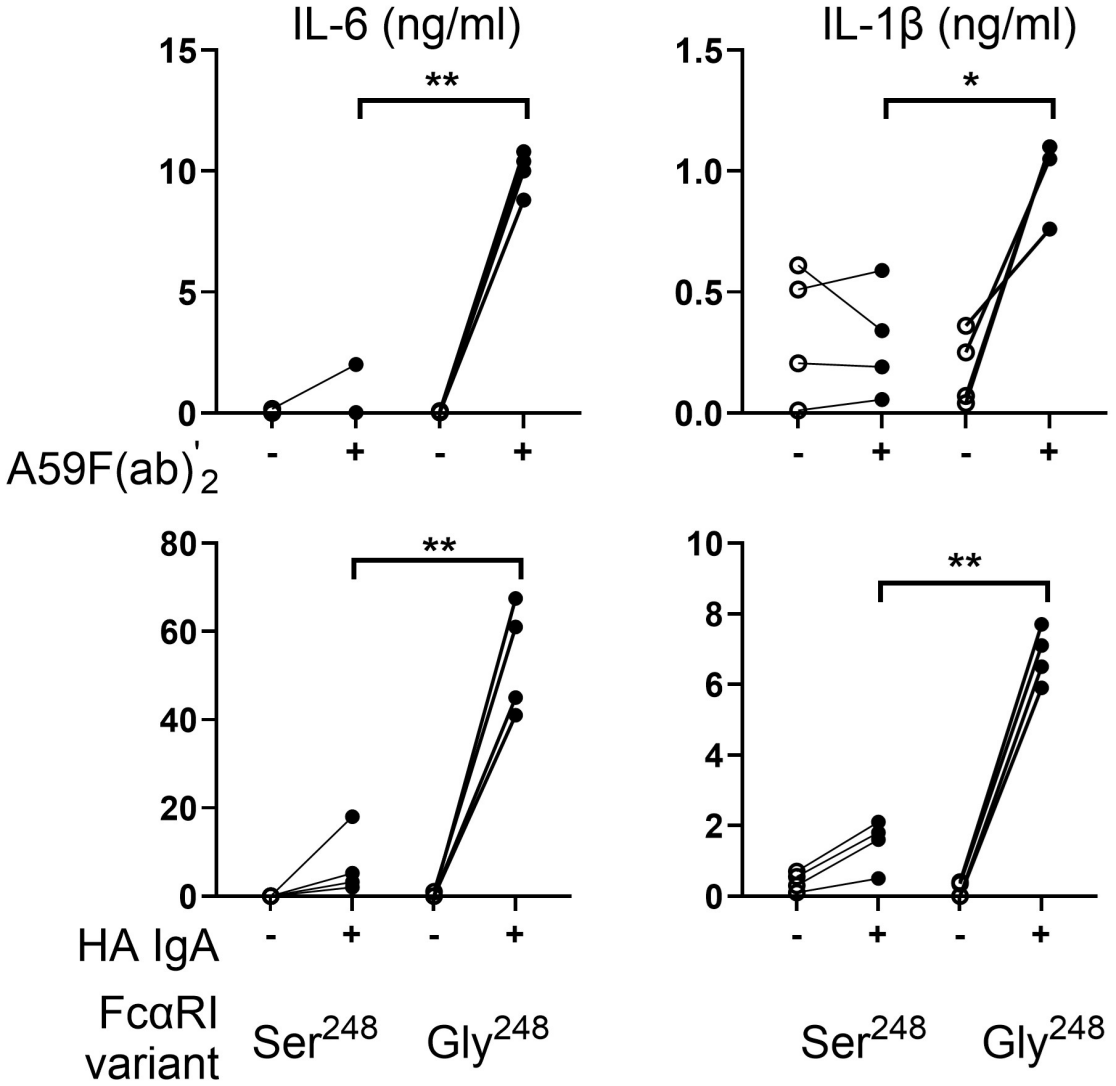
Sequence variation of FcαRI (S^248^G) alters IL-6 and IL-1β production in human monocytes. Peripheral blood monocytes (MNC) (*n* = 4) homozygous for FcαRI Ser^248^ or Gly^248^ variants were stimulated with mAb A59 F(ab′) _2_ or heat-aggregated IgA (HA IgA). IL-6 and IL-1β release were determined by ELISA at 24 h following stimulation. Statistical significance was determined using the paired *t*-test (^*^ *p* < 0.05; ^**^ *p* < 0.01).

We previously reported that a single nucleotide polymorphism (rs16986050; nt 844A>G), which results in a serine- to-glycine change at amino acid 248 in the cytoplasmic domain of FcαRI, significantly alters receptor-mediated functions, including degranulation and cytokine production in cell lines and in primary human neutrophils (25). Because inflammatory cytokines such as IL-1β and IL-6 are mainly monocyte-derived cytokines crucial in effective host defense and the pathogenesis of inflammatory disorders, we investigated the impact of these two natural variants on IL-1β and IL-6 release in human monocytes from four pairs of homozygous donors for FCAR alleles encoding either Ser^248^ or Gly^248^. As predicted, FcαRI Ser^248^ and Gly^248^ variants differed significantly in their ability to signal cytokine production in human monocytes (**Figure 1**). Engagement of FcαRI Gly^248^ with anti-FcαRI mAb A59 F(ab′)_2_ induced significantly higher levels of IL-6 (*p* < 0.01) and IL-1β (*p* < 0.05) compared with FcαRI Ser^248^. Similar results were observed when monocytes were stimulated with heat-aggregated serum IgA (**Figure 1**, lower panels).

**Figure 2:**
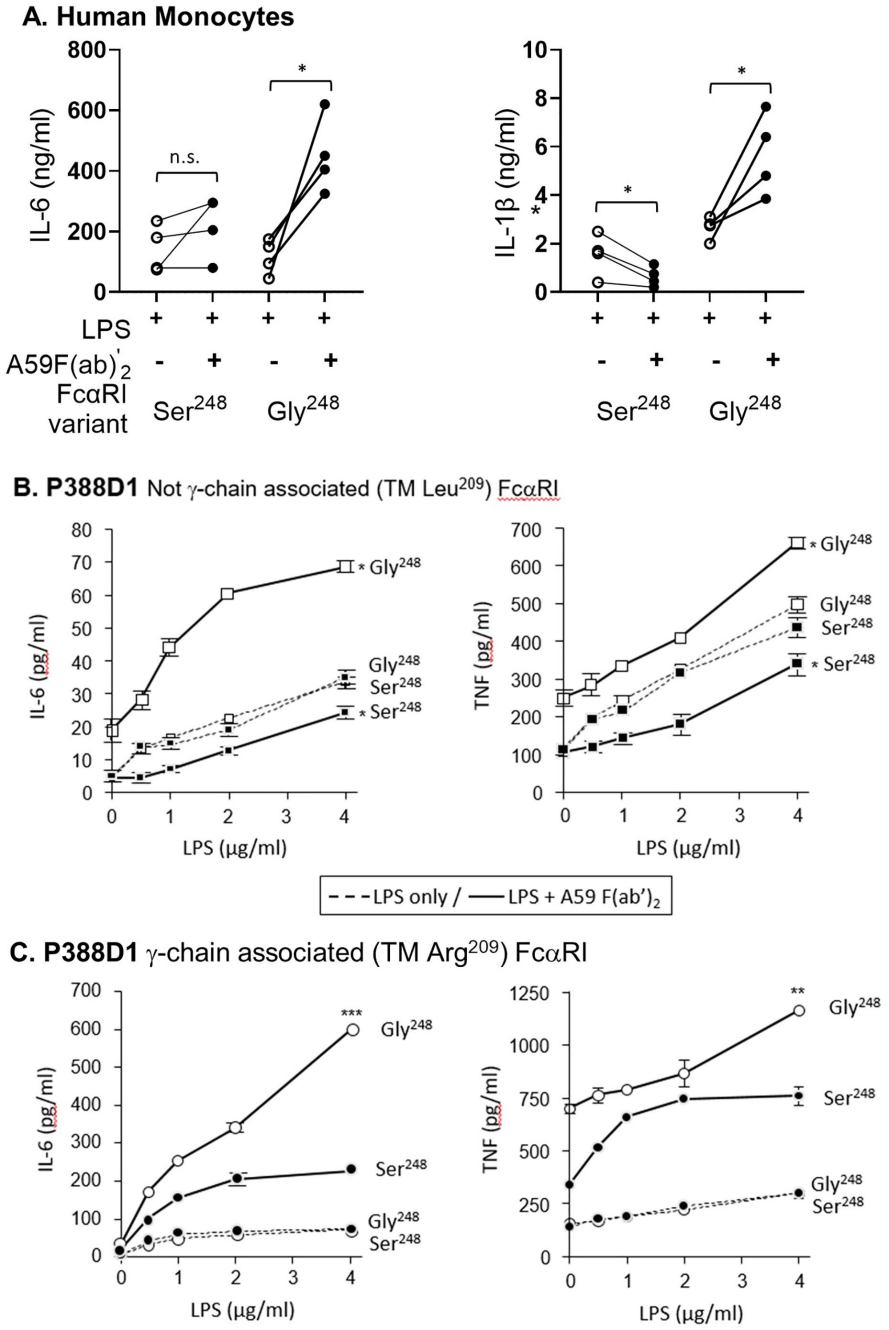
FcαRI Ser^248^ mitigates, while Gly^248^ stimulates, cytokine production. **(A)** Primary monocytes from donors (*n* = 4 pairs) homozygous for FcαRI Ser^248^ or Gly^248^ variants were co-stimulated with LPS and 20 μg/mL A59 F(ab′)_2_. IL-6 and IL-1β released in the media were measured by ELISA at 24 h following stimulation. Statistical significance was determined by a paired *t*-test (^*^ *p* < 0.05; ^**^ *p* < 0.01). **(B, C)** P388D1 murine macrophages stably expressing FcαRI Ser^248^ (filled symbols) or Gly^248^ (open symbols), with or without the transmembrane Arg^209^Leu mutation that disables FcRγ pairing, were stimulated with LPS alone (dashed lines) or with LPS and 20 μg/mL mAb A59 F(ab′) _2_ (solid lines). IL-6 and TNF-α release were measured by ELISA at 24 h following stimulation. **(B)** FcRγ- pairing-disabled (Leu^209^) FcαRI Gly^248^ showed greater cytokine production than Ser^248^, as indicated (mean and standard deviation; ^*^ *p* < 0.05; ^**^ *p* < 0.01, *** *p* < 0.001). **(C)** FcRγ- pairing-enabled (Arg^209^) FcαRI Gly^248^ showed greater cytokine production than Ser^248^, as indicated (^**^ *p* < 0.01). Data shown are representative of three independent experiments giving similar results.

Since some reports suggest that serum IgA may downmodulate inflammatory cytokine release induced by bacteria or LPS stimulation (16, 17), we tested the ability of FcαRI variants to downmodulate LPS-induced responses. Primary monocytes homozygous for FCAR alleles were stimulated with LPS and mAb A59 F(ab′)_2_. FcαRI Ser^248^ inhibited LPS-induced IL-6 and IL-1β release by monocytes stimulated with LPS and A59 F(ab′)_2_ (*p* < 0.01) (**Figure 2A**). In comparison, FcαRI Gly^248^ did not inhibit LPS activity and led to significantly greater IL-6 (*p* < 0.05) and IL-1β (*p* < 0.05) release than LPS alone.

We further characterized the potential of FcαRI Ser^248^ and Gly^248^ to modulate LPS-stimulated cytokine release in P388D1 murine macrophages stably expressing FcαRI alleles with and without FcRγ association. Cells were co-stimulated with LPS and mAb A59 F(ab′)_2_, and the subsequent release of IL-6 and TNF-α was measured. When FcRγ-association was disabled by mutating Arg^209^ to Leu^209^ in the transmembrane domain of FcαRI, FcαRI Ser^248^ stimulation inhibited the LPS-mediated response, as lower levels of IL-6 were released compared to LPS alone (**Figure 2B**). In contrast, FcαRI Gly^248^ enhanced the LPS-activated release of IL-6, despite the absence of FcRγ- chain pairing (**Figure 2B**), supporting an independent activation capacity for this receptor allotype. In the context of FcRγ pairing, both FcαRI Ser^248^ and Gly^248^ variants enhanced the LPS-stimulated release of IL-6, although FcαRI Ser^248^ mediated significantly less activation than FcαRI Gly^248^ (**Figure 2C**). Similar differential modulatory effects of the two FcαRI variants were observed when LPS-stimulated TNF-α was measured (**Figure 2B**). Collectively, these data suggest that the FcαRI α-chain modulates the magnitude, and at times the direction, of FcαRI-mediated anti- or proinflammatory potential. These data further suggest the existence of an α-chain-based regulatory mechanism unique to FcαRI since, unlike other FcRγ-associated FcγR (FcγRI and FcγRIII), FcαRI can be expressed in the absence of FcRγ-pairing (23, 24). Thus, these data demonstrate a mechanism independent of, yet complementary to, the inhibitory FcRγ-ITAM_i_.

### FcαRI Ser^248^ recruits Sab, a Btk signaling inhibitor

**Figure 3:**
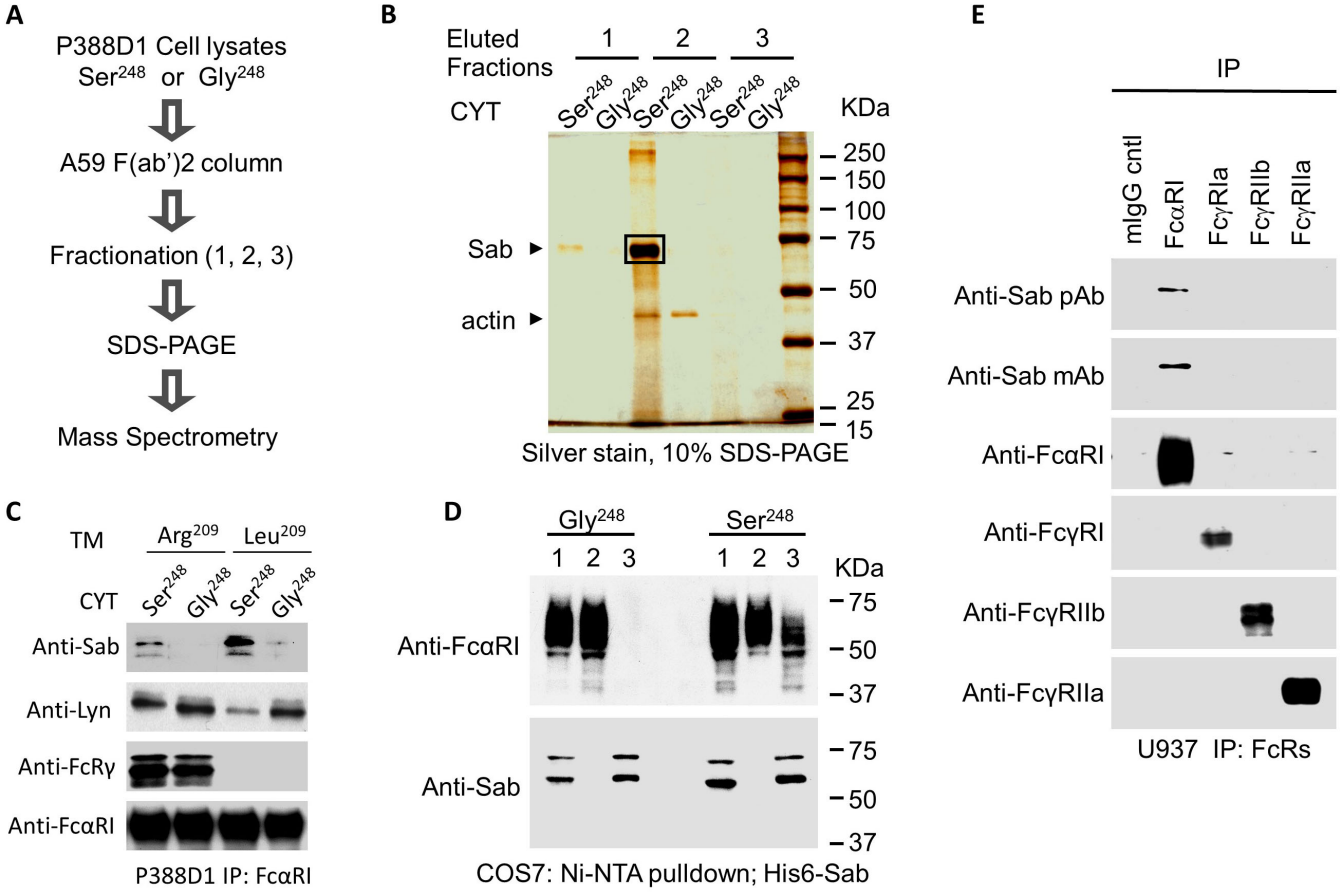
Identification of Sab as an FcαRI-binding protein. **(A)** A workflow diagram showing procedural steps for isolation and identification of FcαRI variant-specific binding proteins. **(B)** FcαRI was immuno-isolated from P388D1 transfectants stably expressing human FcαRI-Ser^248^ or Gly^248^ using an A59 F(ab′)_2_-conjugated Sepharose column. Co-bound proteins were sequentially eluted in three fractions using 0.1% digitonin, pH 7. 2, containing 1 M NaCl (elution fraction 1); 0.1% digitonin, pH 2. 8, plus 0.1 M glycine buffer (neutralized with 1 M Tris·Cl, pH 8.0) (elution fraction 2); and finally, Tris· HCl, pH 6. 8, containing 0.1% SDS and 100 mM DTT (elution fraction 3). The prominent 65- kDa co-eluting protein band was excised from a 10% SDS-PAGE gel and subjected to MALDI– mass spectrometry analysis for identification (**Supplementary Figure 1**). **(C)** FcαRI was immunoprecipitated from P388D1 cells stably expressing the FcαRI Ser^248^ or Gly^248^ variants (CYT), with or without the transmembrane (TM) Arg^209^Leu point mutation that disables association with FcRγ. Co-immunoprecipitates were immunoblotted for Sab (mAb 2B5.1), Lyn (rabbit polyclonal Ab), FcRγ (rabbit polyclonal Ab), and anti-CD89 mAb MIP8a. **(D)**. An Ni–NTA agarose column was used to pull down His-tagged human Sab from COS7 cells co-expressing His6-Sab and the FcαRI-Ser^248^ or Gly^248^ variant. Co-precipitated proteins were separated on 10% SDS-PAGE and immunoblotted for FcαRI and His6-Sab in the input sample (1), the unbound flow- through (2), and the bound (3) fractions. Data shown are representative of four experiments giving similar results. **(E)** FcαRI, FcγRI, FcγRIIb, and FcγRIIa were immunoprecipitated from U937 cells using A59 F(ab′)_2_, 32.2 F(ab′)_2_, IV.3 Fab, and 4F5 F(ab′)_2_ antibodies, respectively. Immunoprecipitates were separated on 10% SDS-PAGE and immunoblotted for Sab (polyclonal Ab E-18 and mAb 2B5.1), FcαRI (mAb MIP8a), FcγRI (rabbit polyclonal Ab 3535), FcγRIIa (mAb 11B6), and FcγRIIb (mAb 3B6).

To probe the molecular basis underlying the S^248^G allele-based differential in activity, we used affinity chromatography to isolate FcαRI-associated allele-specific signaling mediators. Using mAb A59 F(ab′)_2_-based affinity columns, FcαRI-associated proteins were isolated from P388D1 cells stably expressing either FcαRI-Ser^248^ or FcαRI-Gly^248^, each with the engineered Arg^209^ to Leu^209^ transmembrane sequence. Co-precipitating proteins were purified by sequential elution and methanol precipitation, separated on SDS-PAGE, and visualized by silver stain (**Figures 3A, B**). A distinct protein band with an apparent molecular weight of 65 kDa co-precipitated with FcαRI-Ser^248^ but not with FcαR-Gly^248^ (**Figure 3B**). The excised band was identified by MALDI-MS proteomic sequencing as murine Sab (**Supplementary Figure 1**).

To demonstrate that Sab co-precipitated with FcαRI Ser^248^ and establish its immuno-identity, Western blots of FcαRI immunoprecipitates from P388D1 transfectants were probed with a mouse anti-Sab monoclonal antibody (2B5.1). Immunoblots show that Sab co-precipitates FcαRI Ser^248^ but not the Gly^248^ variant (**Figure 3C**). To confirm the allele specificity of the Sab association, we performed additional pull-down experiments. Lysates from COS7 cells co-expressing His6-tagged human Sab with either FcαRI Ser^248^ or FcαRI Gly^248^ were passed over an Ni–NTA column, which binds the His-tag. Input proteins from the whole cell lysates, (1) unbound flow-through proteins, (2) and eluted bound proteins (3) were immunoblotted with anti-Sab and anti-FcαRI. His6-tagged Sab associated with the FcαRI Ser^248^, as both FcαRI Ser^248^ and Sab protein —but not the FcαRI Gly^248^ isoform and Sab protein —co-eluted from the affinity column (**Figure 3D**). The FcαRI Ser^248^ allele also recruited Sab in human cell lines and primary human leukocytes. We observed Sab recruitment by FcαRI Ser^248^ in human primary monocytes and myeloid lines, including U937, THP-1, and HL-60 (**Supplementary Figure 3**). Sab protein did not co-precipitate with FcγRI (CD64), FcγRIIb (CD32B), or FcγRIIa (CD32A) (**Figure 3E**).

We previously reported that FcαRI Gly^248^ preferentially recruits Lyn (25). In the absence of FcRγ pairing, FcαRI Ser^248^ recruits more Sab and less Lyn (**Figure 3C**). Independent of FcRγ pairing, FcαRI Gly^248^ recruits Lyn, but not Sab. Taken together, these data suggest a reciprocal binding between Lyn and Sab to the FcαRI cytoplasmic domain in an allele-dependent fashion.

### FcαRI Ser^248^ recruitment of Sab suppresses receptor-stimulated functions independent of FcRγ pairing

**Figure 4:**
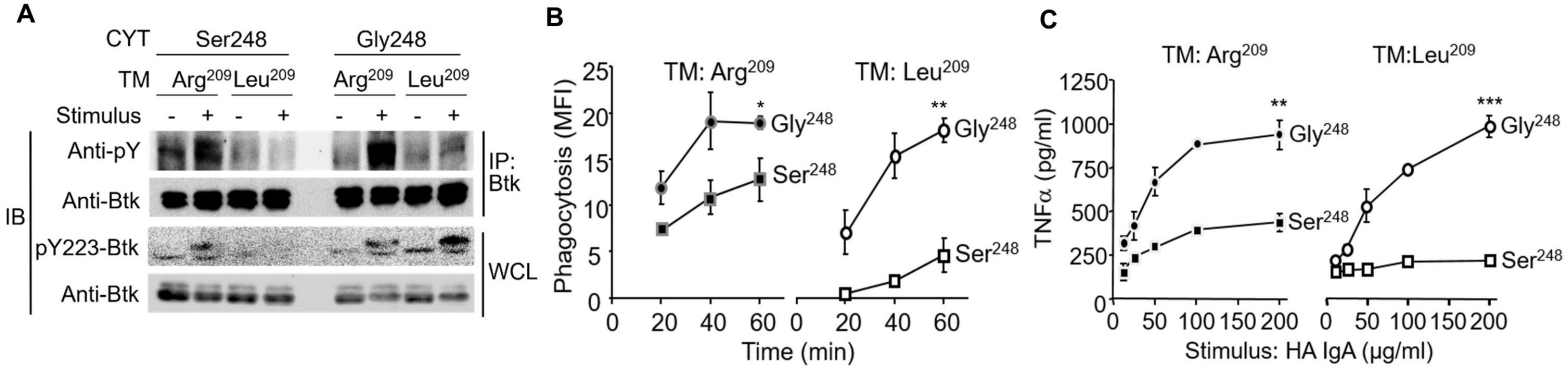
The FcαRI Ser^248^ variant attenuates Btk activation-dependent FcαRI-mediated functions. **(A)** P388D1 cells stably expressing FcαRI Ser^248^ or Gly^248^ variants with or without the Arg^209^Leu transmembrane change, which abrogates FcRγ pairing, were opsonized with A59 F(ab′)_2_ and stimulated with goat antimouse IgG F(ab′)_2_ crosslinking for 3 min. Cells were lysed, Btk was immunoprecipitated, and precipitates were immunoblotted with the antiphosphotyrosine mAb 4G10. Whole cell lysates were also immunoblotted for activated phospho-Btk using the anti-pY223 Btk mAb. **(B)** Transfectant P388D1 cells described above were incubated with A59-opsonized FITC-conjugated Ox RBCs, and phagocytosis of the RBCs was measured using FITC fluorescence (37, 38). Data represent net intracellular MFI after correcting for isotype control (mIgG1-opsonized RBC) fluorescence. Mean and standard deviations are from three independent experiments (^*^ *p* < 0.05; ^**^ *p* < 0.01, paired *t*-test). **(C)** Transfectant P388D1 cells described above were stimulated with increasing concentrations of heat-aggregated IgA (HA IgA), and TNF-α released in the media was measured by ELISA 24 h following stimulation (^**^ *p* < 0.01, ***p < 0.001, paired *t*-test).

Given that Lyn stimulates Btk activation while Sab inhibits Btk activation (34, 50, 51), we tested whether stimulation through FcαRI Ser^248^ and FcαRI Gly^248^ affected site-specific phospho-Btk (pY223), reflecting active Btk, and whether FcRγ pairing was required for these effects, using P388D1 cells expressing FcαRI Ser^248^ and FcαRI Gly^248^ with and without FcRγ pairing. In the P388D1 transfectants, FcαRI Gly^248^ activation of Btk was independent of FcRγ pairing (**Figure 4A**). In contrast, Btk activation by the Ser^248^ allele required FcRγ pairing. Consistent with their differential capacity to activate Btk, which plays a role in phagocytosis (52), FcαRI Gly^248^ mediated robust phagocytosis independent of FcRγ, while FcαRI Ser^248^ mediated only modest phagocytosis of opsonized particles when paired with FcRγ, but downmodulated phagocytosis in the absence of FcRγ pairing (**Figure 4B**). Similar results were observed with TNF-α release in response to heat-aggregated IgA (HA-IgA, **Figure 4C**). Taken together with data on IL-6 release (**Figures 2B, C**), these data suggest that both the FcαRI α-chain and FcRγ play a role in mediating Btk activation and IgA-initiated effector functions. Sab recruitment by FcαRI Ser^248^ suggests that the recruited Sab may modulate Btk activation in both the absence and presence of FcRγ chain pairing, and by extension, in the context of FcαRI Ser^248^Gly heterozygosity. This inhibitory capacity may extend to heterologous receptor systems.

### A truncated Sab incapable of inhibiting Btk reverses FcαRI Ser^248^ inhibitory capacity

**Figure 5:**
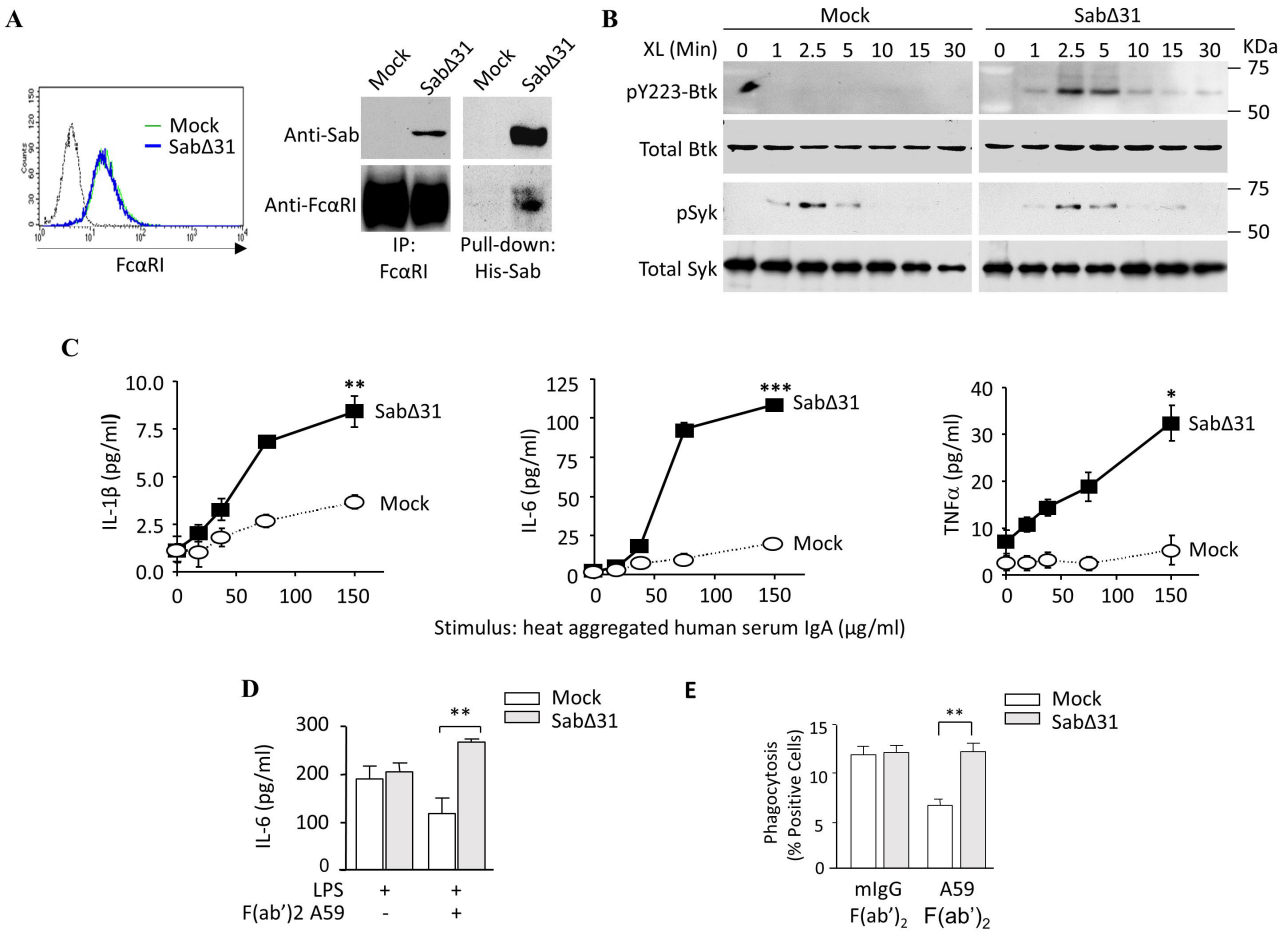
A dominant negative Sab reverses FcαRI S^248^ inhibitory capacity. **(A)** U937 cells stably expressing His6-SabΔ31, which lacks 31 amino acid residues required to bind to and inhibit Btk, were lysed, and FcαRI immunoprecipitated or His6-Sab was pulled down using a nickel Sepharose column. Lysates were separated on 10% SDS-PAGE and immunoblotted for both Sab and FcαRI. **(B)** The U937-SabΔ31 transfectants were opsonized with A59 F(ab′)_2_ and stimulated with GAM F(ab′)_2_ cross-linking for the indicated times. SDS-PAGE separated whole cell lysates were immunoblotted for phospho-Btk, total Btk, and total Syk. To assess phospho-Syk, phosphorylated proteins were immunoprecipitated using PY20-adsorbed beads and immunoblotted for phospho-Syk. **(C)** U937 cells stably expressing SabΔ31 were stimulated with increasing concentrations of human serum IgA for 24 h, and the IL-1β, IL-6, and TNF-α released were measured using ELISA (^**^ *p* < 0.01, *** *p *< 0.001, paired *t*-test). **(D)** SabΔ31-expressing U937 cells were stimulated with 1 µg/mL LPS and A59 F(ab′)_2_ for 24 h. IL-6 produced was measured by ELISA (^**^ *p* < 0.01, unpaired *t*-test). **(E)** SabΔ31-expressing U937 cells were opsonized for 30 min with A59 F(ab′)_2_ and incubated with human IgG-opsonized FITC-conjugated ox RBC for 1 h. Cells were then treated with Trypan Blue, and internalized RBCs were measured as intracellular FITC fluorescence as described above. Data are expressed as percent positive cells following isotype control correction. Mean and standard deviation were obtained from three independent experiments (^**^ *p* < 0.01, paired *t*-test).

Our data suggest that FcαRI Ser^248^ mitigates effector functions by recruiting Sab, which inhibits Btk activation and its downstream signaling cascade. To address whether Sab mediates FcαRI Ser^248^ effects, we stably expressed a dominant-negative form of Sab lacking 31 amino acid residues, which include a Kinase Interacting Motif (53) required for Btk binding (SabΔ31) (34). In U937 cells, which express the FcαRI Ser^248^ variant and FcRγ endogenously, transfection with SabΔ31 did not affect FcαRI expression, and the SabΔ31 construct bound to the Ser^248^ variant (**Figure 5A**). However, compared to mock transfectants, SabΔ31 transfectants failed to inhibit Btk activation, assessed as phospho-Btk, in Btk immunoprecipitates of HA- IgA-stimulated cells (**Figure 5B**). Notably, Syk activation was unaffected (**Figure 5B**). Consistent with an inability to inhibit Btk, SabΔ31 FcαRI Ser^248^ cells produced significantly more IL-1β, IL-6, and TNF-α than mock transfectants (**Figure 5C**). FcαRI Ser^248^ stimulation in SabΔ 31-transfected cells resulted in enhancement of both LPS-stimulated IL-6 production (**Figure 5D**) and IgG-mediated phagocytosis (**Figure 5E**). These data indicate that FcαRI Ser^248^ inhibits activation by recruiting Sab, which inhibits Btk activation, and that SabΔ31 fails to inhibit Btk, reversing the FcαRI Ser^248^ inhibitory phenotype.

### Phosphorylation of FcαRI Ser^248^ by CK1δ enhances the recruitment of Sab

**Figure 6:**
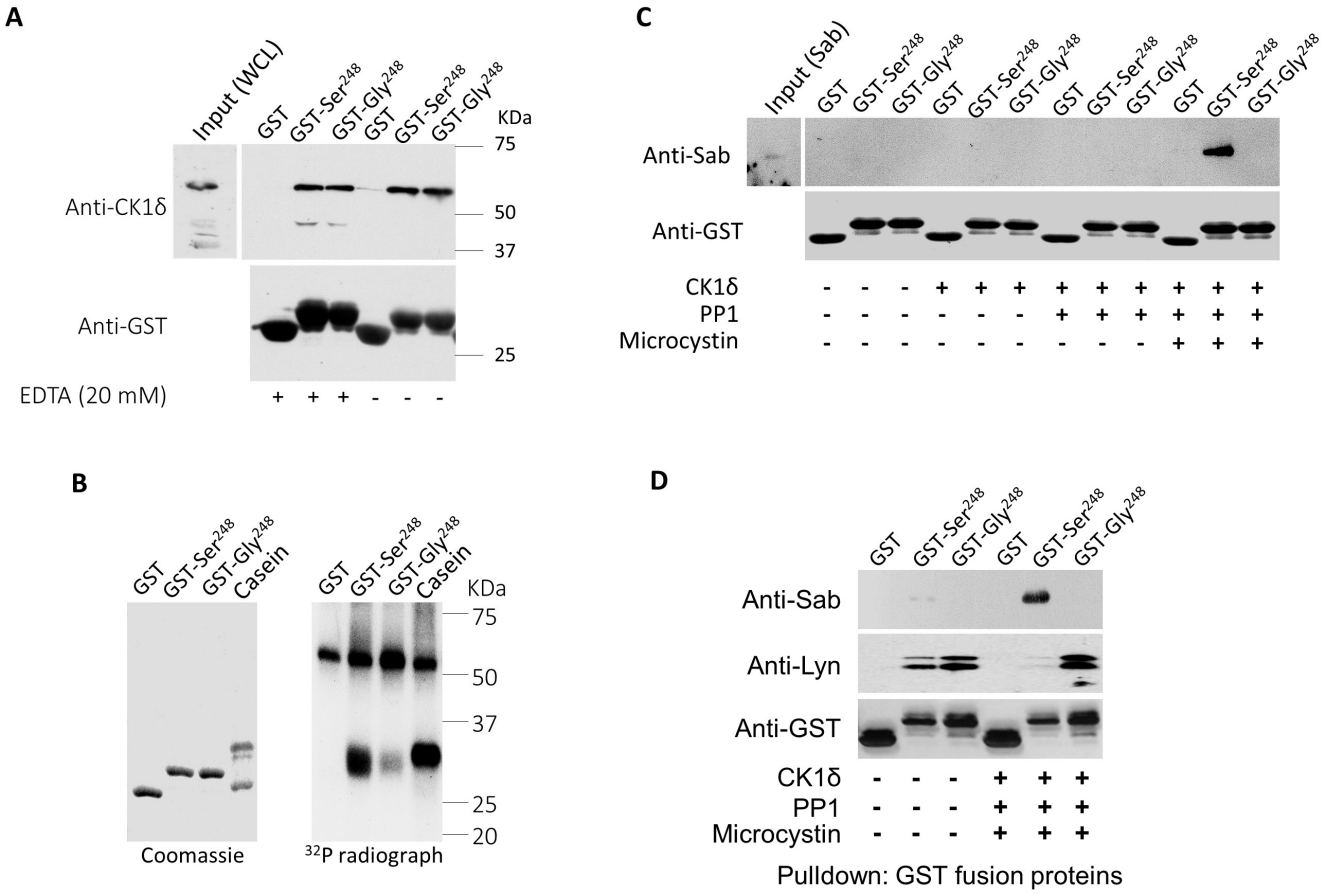
CK1δ-phosphorylated FcαRI Ser^248^ modulates Sab binding. **(A)** Immobilized GST or GST fusions to the cytoplasmic domain (CYT) Ser^248^ or Gly^248^ variant of FcαRI were incubated with lysate from U937 cells. Co-precipitating proteins were SDS-PAGE separated and immunoblotted for CK1δ. **(B)** Immobilized GST or GST fusions to the FcαRI CYT variants were incubated with purified activated CK1δ and ^32^P-ATP for 20 min at room temperature. Proteins were separated on 10% SDS-PAGE, and the gel was dried and exposed to film. Casein was used as a positive control for CK1δ activity. **(C)** GST or GST fusions to the FcαRI CYT variants were treated with CK1δ and incubated with lysate from U937 cells expressing His6-Sab. SDS-PAGE- separated co-precipitating proteins were probed for His6-Sab. **(D)** Immobilized GST or GST fusions to FcαRI CYT variants were treated with activated CK1δ and incubated with lysate from U937 cells expressing His6-Sab. SDS-PAGE- separated co-precipitating proteins were immunoblotted for Sab and Lyn kinase.

Our data indicate that FcαRI Gly^248^ binds Lyn kinase, while Ser^248^ preferentially recruits Sab in a reciprocal fashion (**Figure 3C**). To explore the molecular basis of the ability of Ser^248^ to bind both Sab and Lyn reciprocally, we first examined the protein sequence surrounding the Ser/Gly^248^ variants to identify known motifs. Alignment of this region of FcαRI from several species suggests that Ser^248^ is within a conserved unconventional SH3-domain binding motif, W^247^xxQ^250^, which may support binding of SH3- domain-containing molecules like Lyn and Sab (**Supplementary Figure 4**). In addition, the alignment identified a second conserved motif, E^237^xxxD/E^241^xxE/D^244^xxxS^248^, a nonphosphate-directed casein kinase 1 (CK1) binding site with Ser^248^ downstream of negatively charged residues (54). Since FcαRI Ser^248^ recruits Sab, we hypothesized that CK1δ binds to the FcαRI CK1 motif to phosphorylate Ser^248^, which in turn facilitates Sab binding. First, we demonstrated that purified GST-FcαRI cytoplasmic domain fusion proteins (GST-CYT), but not GST alone, could pull down CK1δ from U937 cell lysates (**Figure 6A**). Using allele-specific GST-CYT fusion proteins, we demonstrated that CK1 isoforms could phosphorylate FcαRI Ser^248^ robustly *in vitro* (**Figure 6B**). Activated CK1δ (and CK1ϵ but not CK1α, CKγ1, CKγ2, or CKγ3; data not shown) could efficiently phosphorylate FcαRI CYT at Ser^248^, with only minimal phosphorylation of the Gly^248^ construct.

### Phosphorylation of FcαRI Ser^248^ promotes Sab binding but disrupts Lyn binding

We next examined whether phosphorylation of FcαRI Ser^248^ is required for Sab binding. Using His-tagged Sab and GST-CYT-Ser^248^ with or without CK1δ treatment in a pull-down assay, we showed that Sab binds to FcαRI CYT-Ser^248^ after treatment with PP1-activated CK1δ (**Figure 6C**), confirming that phosphorylation of Ser^248^ is required for enhanced Sab binding. Given that Lyn is also recruited by FcαRI, and that Sab recruitment by the Ser^248^ variant requires prephosphorylation, we tested whether Lyn recruitment also requires prephosphorylation of the cytoplasmic tail. GST-CYT fusions were treated with PP1-activated CK1δ and incubated with lysates from U937 cells, which express Lyn endogenously and stably express His-tagged Sab. Blots of proteins co-precipitating with the GST constructs, probed for Lyn and Sab, showed that Lyn kinase was associated with both Ser^248^ and Gly^248^ alleles before treatment with CK1δ, albeit more robustly with the Gly^248^ allele (**Figure 6D**). However, following CK1δ treatment, Lyn was no longer associated with Ser^248^, and Sab recruitment increased (**Figure 6D**). These data indicate a Ser^248^ phosphorylation-dependent reciprocal binding of Sab and Lyn and position Sab recruitment by Ser^248^ as an intrinsic inhibitory mechanism of both FcαRI-induced and other heterologous receptor-mediated cell activation.

**Figure 7:**
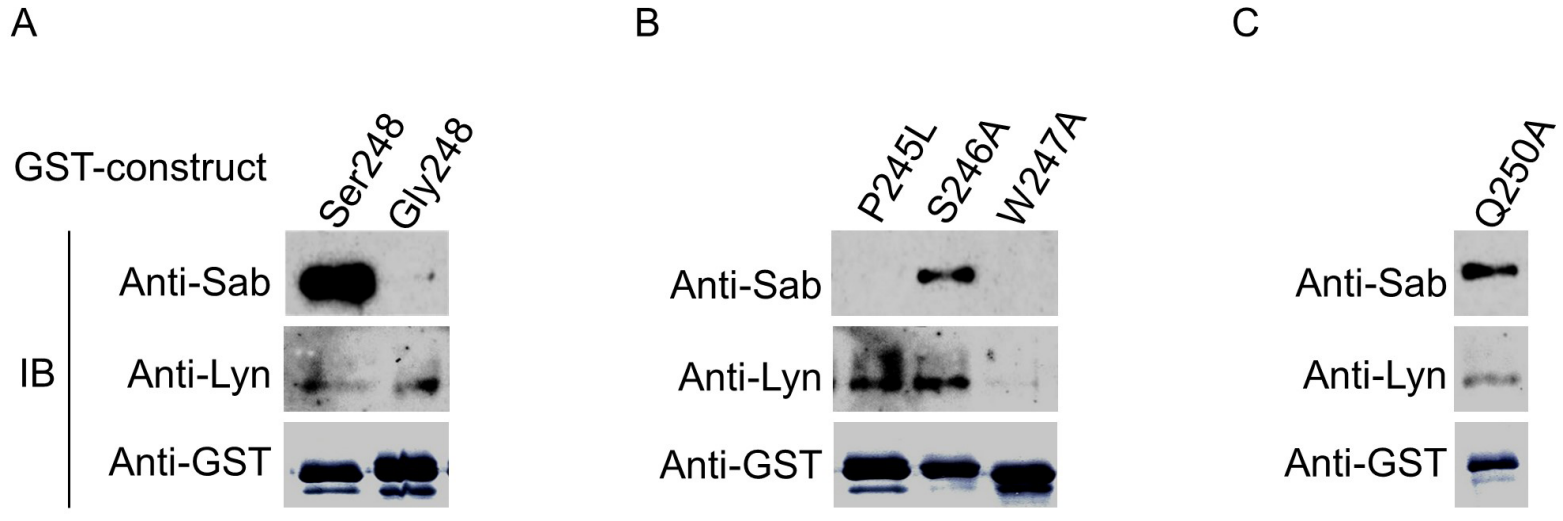
Conserved amino acid residues in a putative atypical SH3 domain in FcαRI CYT alter reciprocal recruitment of Sab and/or Lyn. **(A)** GST-FcαRI Ser^248^ or GST-FcαRI Gly^248^ CYT fusion proteins were treated with CK1δ, then incubated with lysates from U937 cells expressing His6-Sab. Co-precipitating proteins were SDS-PAGE separated and immunoblotted for His6-Sab and Lyn kinase. **(B, C)** GST-FcαRI Ser^248^ fusion proteins carrying point mutations within a putative atypical SH3 domain were treated with CK1 δ, then incubated with lysates from U937 cells expressing His6-Sab. Co-precipitating proteins were SDS-PAGE separated and immunoblotted for His6-Sab and Lyn kinase.

Finally, since S^248^ and the preceding W^247^ in the unconventional SH3-binding motif are conserved across several different species (**Supplementary Figure 4**), we examined whether these and other residues in the motif played a role in FcαRI cytoplasmic domain binding of Sab and Lyn. We created point mutations within our GST-CYT-Ser^248^ cytoplasmic domain construct, phosphorylated the mutant fusion proteins with activated CK1δ, and then incubated the phosphorylated mutant proteins with cell lysate from His6-Sab-expressing U937. As shown in **Figure 7**, compared with the S248 WT, the P245L (a rare variant) and W247A changes disrupt Sab binding, confirming a critical requirement for these residues in Sab recruitment by FcαRI (**Figure 7**). In contrast, compared to the S248 WT, the S246A change reduced but did not eliminate Sab recruitment. While the W247A change effectively eliminated Lyn recruitment, the P245L and S246A had little or no effect on Lyn recruitment. The Q250A change significantly reduced but did not eliminate Lyn binding (**Figure 7**). Taken together, these data suggest that both Sab and Lyn interact with residues in and around the W^247^xxQ^250^ unconventional SH3-domain binding motif in the FcαRI cytoplasmic domain and that critical residues in the sequence motif have differential effects on Sab and Lyn binding, resulting in reciprocal recruitment of the two proteins (**Supplementary Figure 5**). Of interest, neither the canonical binding site for PKC in FcγRIIIa nor the site for CK2 in FcγRIa cytoplasmic domains —each of which alters function when mutated experimentally (55–60)—has nonsynonymous SNPs in large population studies (**Supplementary Figure 2**). Similarly, the germline sequences for the ITAM and ITIM motifs in the two *FCGR2A* and *FCGR2B* genes are monomorphic (**Supplementary Figure 2**, legend), underscoring the uniqueness of having both activating and inhibitory alleles in the α-chain of FcαRI.

## Discussion

Fc receptors transduce activation signals by means of ITAMs located either in the cytoplasmic domain of their ligand-binding chain (e.g., FcγRIIa) or in the associated FcRγ chain (e.g., FcαRI, FcγRI, FcγRIIIa). Multivalent cross-linking of the receptors results in tyrosine phosphorylation of the ITAMs, recruitment and phosphorylation of Syk, and subsequent phosphorylation of downstream signaling intermediates, resulting in cell effector functions (1, 61–63). Counter-balancing cell activation has been the role of the immunoreceptor tyrosine-based inhibitor motif (ITIM) in the inhibitory receptor, FcγRIIb (64). Upon cross-linking and consequent tyrosine phosphorylation, ITIMs recruit SHP and SHIP phosphatases, which dephosphorylate signaling intermediates and inhibit cell effector functions (64).

For decades, experimental reports have noted that serum IgA can exhibit seemingly paradoxical inflammatory and anti-inflammatory properties (9–17). These findings were particularly confounding, since there is no ITIM-containing receptor for IgA comparable to FcγRIIb. Monteiro’s group defined a functionally inhibitory ITAM configuration (ITAM_i_) as an inhibitory mechanism initiated by low levels of receptor oligomerization (20, 65, 66). With low, often dimeric, cross-linking of receptors, differential recruitment and site-specific phosphorylation of SHP-1 at Tyr536 by Lyn can function to inhibit active signaling (ITAM_i_) (20, 65–67). Interestingly, however, FcαRI does not require an associated FcRγ for expression and is often not associated with FcRγ in primary cells (23, 24).

We have defined an FcαRI allele-specific inhibitory mechanism independent of FcRγ-chain pairing and not involving either ITAM_i_ or an ITIM. In the absence of FcRγ-chain pairing, the FcαRI Ser^248^ variant downmodulates both intrinsic signals and heterologous activation signals involving receptor cross-talk by recruiting Sab, a trans-inhibitor of Btk activation, which is central to receptor signaling. In contrast, the FcαRI Gly^248^ variant does not bind Sab. Constitutively, FcαRI Gly^248^ recruits Lyn kinase and delivers activation signals with oligomerization in both the absence and the presence of FcRγ-chain pairing. While the FcαRI Ser^248^ cytoplasmic domain mitigates activation signals by recruiting Sab, a trans-inhibitor of Btk, in a serine phosphorylation-dependent fashion mediated by CK1δ, low-level cross-linking of the FcαRI Ser^248^ allotype likely initiates an ITAM_i_ as well, if the ligand-binding α-chain is paired with the FcRγ chain. This complementarity of functions may be particularly important since FcαRI, unlike FcγRs, is expressed in the absence of the FcRγ chain (23, 24). Even in the context of higher-order cross-linking with activating ITAM-mediated signals, the FcαRI Ser^248^ allele is able to downmodulate the intensity of those activating signals (**Figure 4C**). Thus, in addition to ITAM_i_, a genetically determined, allele-based, and phosphorylation-dependent mechanism provides a non-ITAM molecular mechanism underlying the range —and at times, divergent —FcαRI functional phenotypes.

We, and others, have documented that the cytoplasmic domains of Fcγ receptor ligand-binding α-chains, which signal through the associated FcRγ-ITAM, have canonical serine/threonine phosphorylation sites. Modulation of the receptor complex signaling in a phosphorylation-dependent fashion has been demonstrated through site-specific mutation studies (55–60). However, none of these canonical sites are allelic in human populations (**Supplementary Figure 2**), and none has demonstrated the ability to alter signaling in the absence of FcRγ-chain pairing. The FcαRI system is unique among FcR both in the ability of the α-chain to be expressed and to signal in the absence of FcRγ-chain pairing, and in having the properties of activation and inhibition incorporated as alleles rather than as separate ITAM- and ITIM-containing genes.

A central role for Btk in immune receptor signaling has long been observed (68). Btk plays a role in B-cell receptor maturation and signaling (69, 70), in mast cell FcεRI signaling (71), and in phagocyte FcγR signaling (68, 72–75). Monteiro’s group and others have previously shown that Btk is involved in myeloid cell FcαRI signaling (61, 76), and recent observations indicate that Btk plays an important role in cytokine production and phagocytosis (52). Sab binding to the SH3 domain of Btk and its subsequent inhibition of Btk phosphorylation and activation have also been documented (34). Our results here show, for the first time, that the FcαRI Ser^248^ allele directly recruits Sab, which binds to and inhibits Btk activation. That Sab blunts FcαRI activation by inhibiting Btk is supported by our use of a dominant-negative Sab, which is incapable of inhibiting Btk and which reverses the FcαRI Ser^248^ inhibitory phenotype.

We have identified an SH3-like binding motif (PSWS^248^QQ) in the FcαRI cytoplasmic domain to which Sab and Lyn bind in a reciprocal fashion. Our mutational analysis indicates that specific amino acids residing within the motif have differential effects on the binding of each protein. While Sab binding is serine phosphorylation-dependent and Lyn binding is not, the complete mechanism of reciprocal recruitment, including the temporal dynamics of the serine modification by CK1δ and the identity of the phosphatase that dephosphorylates Ser^248^, is currently unclear. Nevertheless, our results have revealed multiple levels in the regulation of FcαRI signaling. The germline variant encoding the Ser^248^Gly polymorphism modulates signal intensity and, in the absence of FcRγ-chain pairing, may result in allele-dependent divergent receptor functional phenotypes. Resting neutrophils express little or no FcRγ, but in the presence of some cytokines, including IFN-γ (1, 24, 77), FcRγ protein expression is induced, with a likely increase in the proportion of FcαRI chains that are paired with FcRγ. In the presence of such chain pairing and oligovalent ligands, an inhibitory ITAM_i_ may be induced as a second down-modulatory mechanism. Taken together with the predominant Ser^248^ allele, IgA may serve as a “silent housekeeper” in dampening immune system activity (78). A more intense cytokine milieu and multivalent ligands would likely overcome these inhibitory mechanisms. Each of these mechanisms has implications not only for genetic contributions to disease severity (27) but also for the use of IgA heavy chains in the development of antibody-based therapeutics (28–32, 78).

## Data Availability

The datasets presented in this study can be found in online repositories. The names of the repository/repositories and accession number(s) can be found in the article/**Supplementary Material**.

## Supplementary material

Supplementary Figure 1 MALDI/MS identifies Sab (SH3BP5) association. A 65KDa distinct protein that co-purified specifically with FcαRI Ser^248^ was subjected to MALDI-MS analysis (35). The resulting peptide sequences identified the protein as murine SH3BP5 (Sab).

Supplementary Figure 2 Non-synonymous cytoplasmic domain germline variants for IgA and IgG antibody receptors. Whole exome sequencing was performed on 983,578 donors, and variants were called and filtered for non-synonymous single nucleotide polymorphisms in the cytoplasmic domains of IgA and IgG antibody receptors as described in Methods. FCAR, the gene encoding FcαRI, has as a common SNP (rs16986050) with minor allele (Gly^248^) frequencies of ~0.18 in European Americans, ~0.30 in African Americans, ~0.07 in East Asians and ~0.05 in Mexicans (39). There are some references to a putative non-synonymous SNP in the cytoplasmic domain of FcγRIIa encoding Leu ^273^Pro with a potential impact on receptor function (79, 80). PacBio circular consensus sequencing with phased assembly of the FCGR region in 402 chromosomes revealed that ^273^Pro was encoded only in the chimeric FcγR2a/FcγR2c protein in the setting of an 80 kb deletion of FCG3A (N=6).

Supplementary Figure 3 Sab coimmunoprecipitates with FcαR1 Ser^248^-bearing cell lines and primary cells. Sab coimmunoprecipitates with FcαRI from FcαRI Ser^248^-bearing human monocytic cell lines U937, THP-1 and HL-60, and from peripheral blood neutrophils (PMN) and mononuclear cells (MNC). Cells were opsonized with A59 F(ab’)_2_ and expressed FcαRI immunoprecipitated (IP) with goat anti-mouse IgG-beads. FcαRI from neutrophils intracellular pools (PMN, lane 8) was precipitated using F(ab’)_2_ A59-beads. Immunoprecipitates were separated by SDS-PAGE and immunoblotted for Sab. An immunoblot of purified Sab (His6-Sab) with or without PP1 dephosphorylation (left insert) shows several Sab species of different electrophoretic mobility as observed in cell lines and primary cells suggesting that post-translationally modified forms of Sab likely exist within cells. Data are representatives of three to five independent experiments giving similar results.

Supplementary Figure 4 Alignment of the human FcαRI cytoplasmic domain amino acid sequence with homologues from different species reveals a conserved “WSxQ” motif for Lyn/Sab interaction. A proline residue precedes the conserved “W^247^S^248^xQ^250”^ motif in the FcαRI cytoplasmic domain and resembles an unconventional SH3-domain binding motif known to support the binding of Lyn or other SH3-domain containing molecules. The indicated “E^237^xxxE/D^241^xxD/E^244^xxW^247^S^248^” motif resembles a non-phosphorylation-directed CK1-targeting site. A series of positively charged residues in CK1δ and CK1ϵ which are flanked by hydrophobic residues align well with corresponding negatively charged residues in the CK-1 targeting site. Ser^248^ is indicated by a red arrowhead. Amino acid resides that are conserved across species or which maintain positional charge are highlighted in yellow.

Supplementary Figure 5 Model for a genetically determined, serine-based and phosphorylation-dependent switch that modulates the signaling capacity of FcαRI. The naturally occurring FcαRI-Ser^248^ is phosphorylated by CK1δ which enhances recruitment of SH3BP5 (Sab), a Btk inhibitor, diminishes recruitment of Lyn kinase, and inhibits Btk-mediated FcαRI receptor signaling (left panel). Dephosphorylation of Ser^248^ or replacement by the naturally occurring Gly^248^ genetic variant diminishes Sab recruitment, enhances recruitment of Lyn kinase, and enables activation of receptor signaling (right panel).
