# Supplementary figures and images for: A genetically determined molecular switch modulates the anti-inflammatory potential of human IgA
Source: Front Immunol. 2025 Aug 27;16:1641351. doi: 10.3389/fimmu.2025.1641351 (PMC12420274; doi:10.3389/fimmu.2025.1641351)

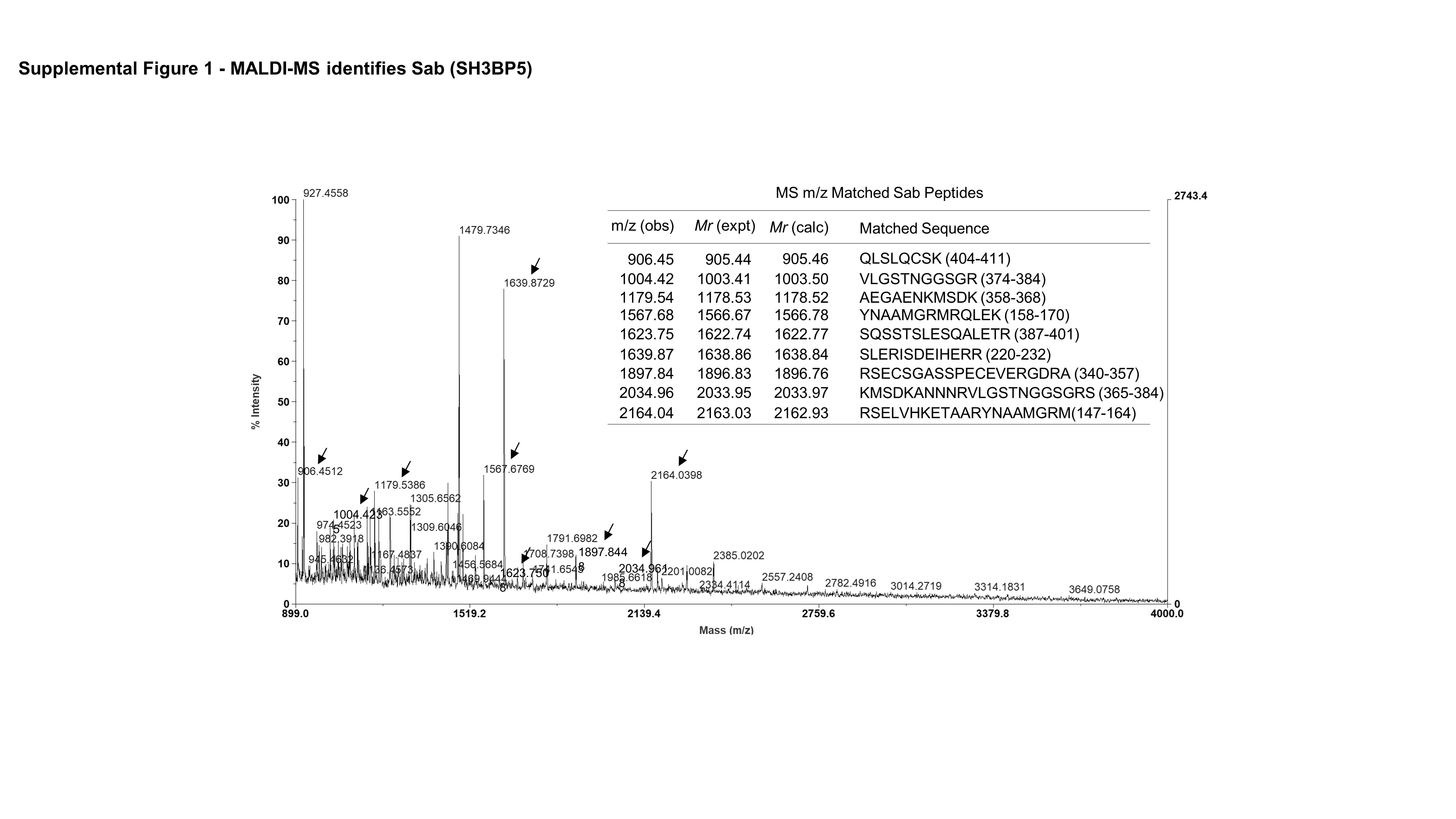

Supplement: Supplementary Figure 1 — MALDI/MS identifies Sab (SH3BP5) association. A 65KDa distinct protein that co-purified specifically with FcαRI Ser248 was subjected to MALDI-MS analysis (35). The resulting peptide sequences identified the protein as murine SH3BP5 (Sab). [file Image1.jpeg]

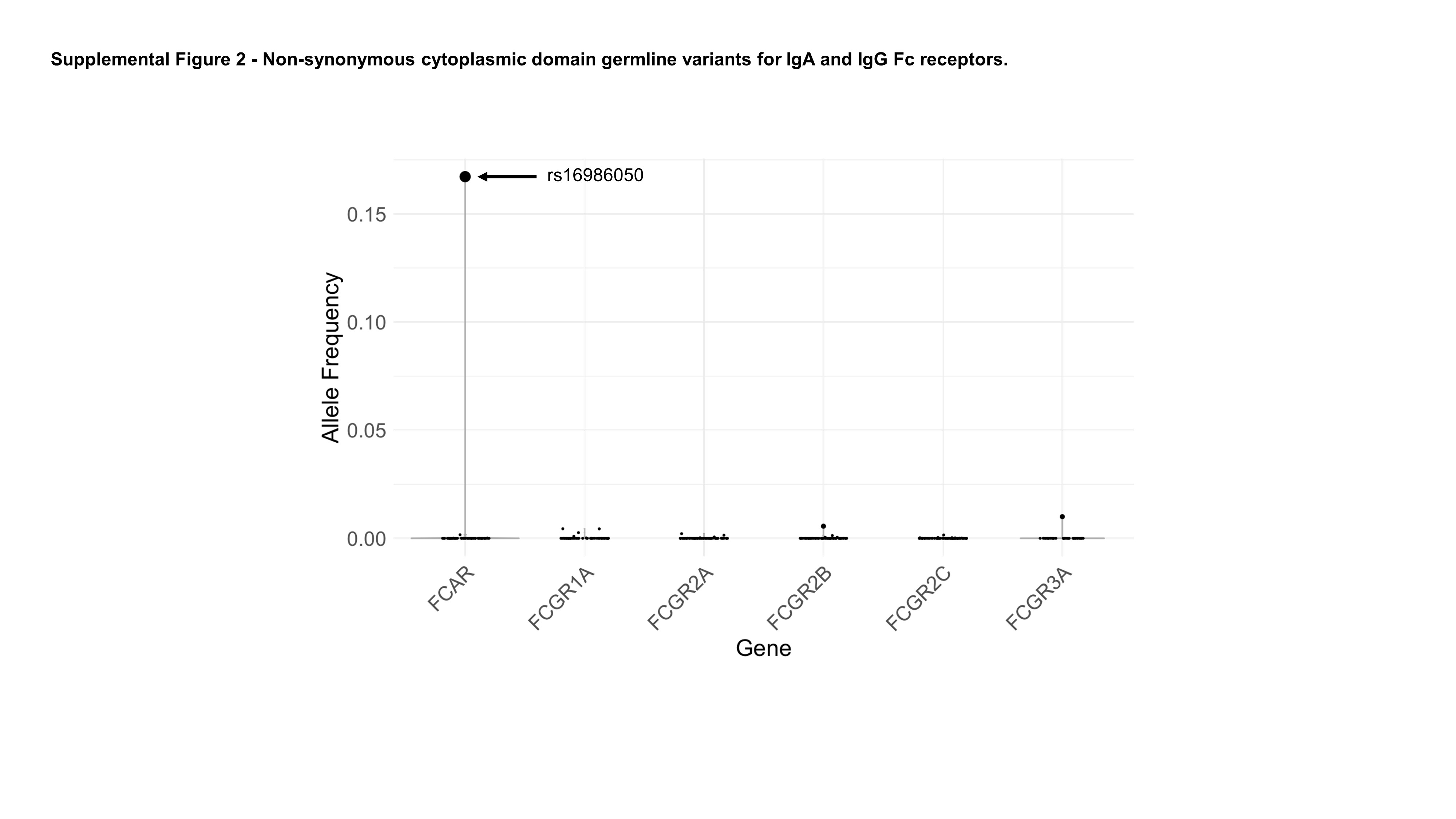

Supplement: Supplementary Figure 2 — Non-synonymous cytoplasmic domain germline variants for IgA and IgG antibody receptors. Whole exome sequencing was performed on 983,578 donors, and variants were called and filtered for non-synonymous single nucleotide polymorphisms in the cytoplasmic domains of IgA and IgG antibody receptors as described in Methods. FCAR, the gene encoding FcαRI, has as a common SNP (rs16986050) with minor allele (Gly248) frequencies of ~0.18 in European Americans, ~0.30 in African Americans, ~0.07 in East Asians and ~0.05 in Mexicans (39). There are some references to a putative non-synonymous SNP in the cytoplasmic domain of FcγRIIa encoding Leu 273Pro with a potential impact on receptor function (79, 80). PacBio circular consensus sequencing with phased assembly of the FCGR region in 402 chromosomes revealed that 273Pro was encoded only in the chimeric FcγR2a/FcγR2c protein in the setting of an 80 kb deletion of FCG3A (N=6). [file Image2.jpeg]

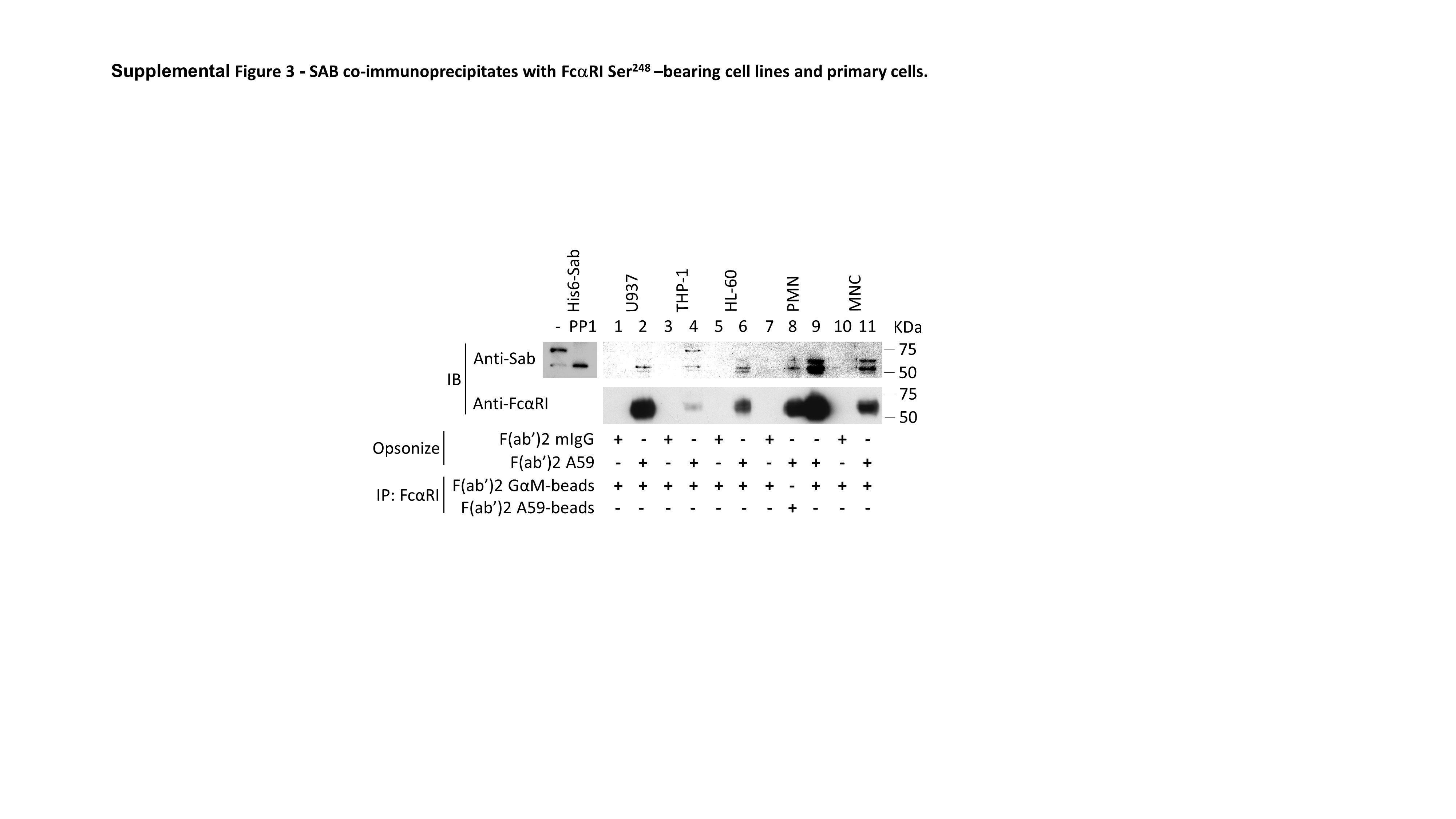

Supplement: Supplementary Figure 3 — Sab coimmunoprecipitates with FcαR1 Ser248-bearing cell lines and primary cells. Sab coimmunoprecipitates with FcαRI from FcαRI Ser248-bearing human monocytic cell lines U937, THP-1 and HL-60, and from peripheral blood neutrophils (PMN) and mononuclear cells (MNC). Cells were opsonized with A59 F(ab’)2 and expressed FcαRI immunoprecipitated (IP) with goat anti-mouse IgG-beads. FcαRI from neutrophils intracellular pools (PMN, lane 8) was precipitated using F(ab’)2 A59-beads. Immunoprecipitates were separated by SDS-PAGE and immunoblotted for Sab. An immunoblot of purified Sab (His6-Sab) with or without PP1 dephosphorylation (left insert) shows several Sab species of different electrophoretic mobility as observed in cell lines and primary cells suggesting that post-translationally modified forms of Sab likely exist within cells. Data are representatives of three to five independent experiments giving similar results. [file Image3.jpeg]

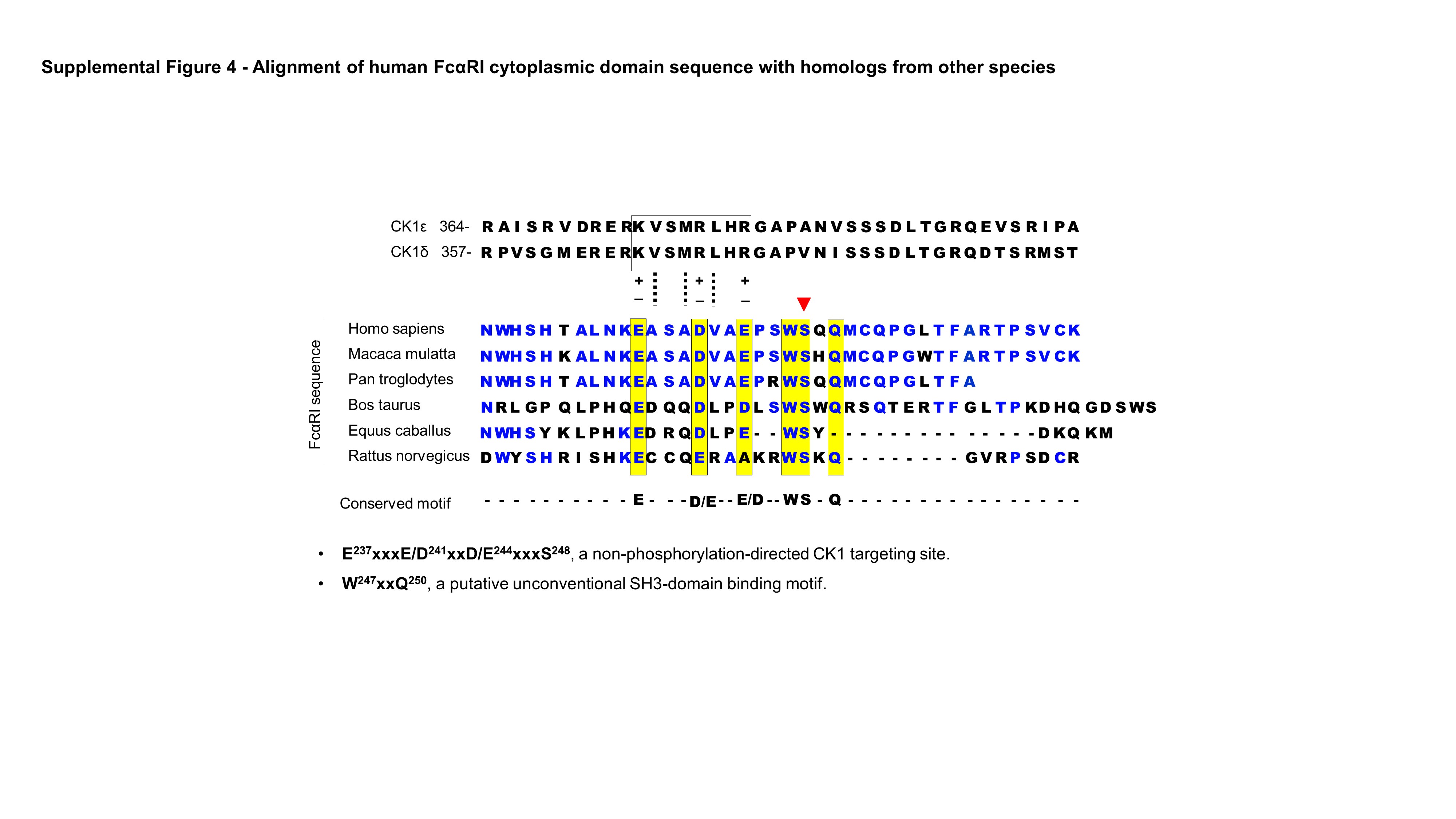

Supplement: Supplementary Figure 4 — Alignment of the human FcαRI cytoplasmic domain amino acid sequence with homologues from different species reveals a conserved “WSxQ” motif for Lyn/Sab interaction. A proline residue precedes the conserved “W247S248xQ250” motif in the FcαRI cytoplasmic domain and resembles an unconventional SH3-domain binding motif known to support the binding of Lyn or other SH3-domain containing molecules. The indicated “E237xxxE/D241xxD/E244xxW247S248” motif resembles a non-phosphorylation-directed CK1-targeting site. A series of positively charged residues in CK1δ and CK1ϵ which are flanked by hydrophobic residues align well with corresponding negatively charged residues in the CK-1 targeting site. Ser248 is indicated by a red arrowhead. Amino acid resides that are conserved across species or which maintain positional charge are highlighted in yellow. [file Image4.jpeg]

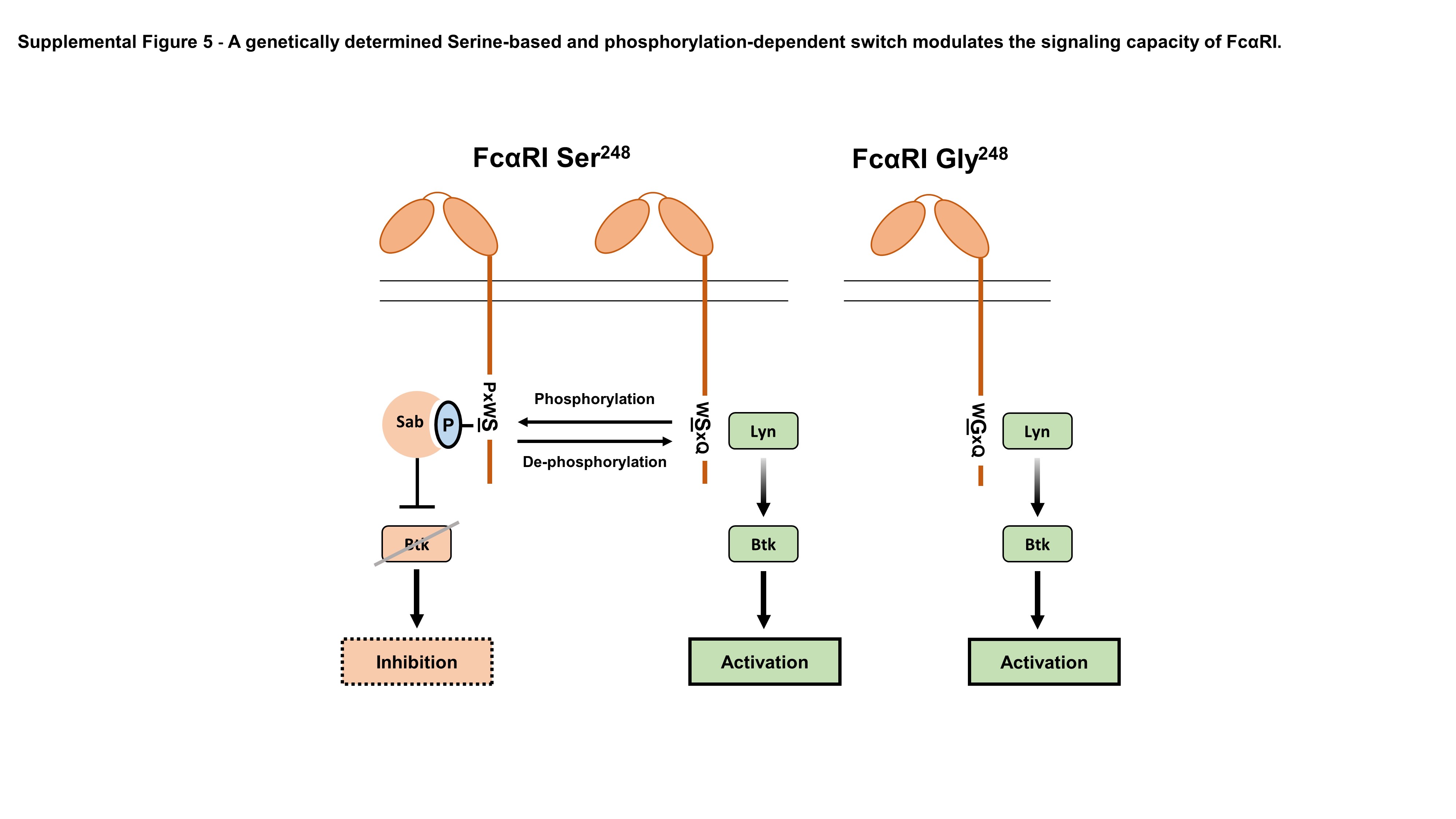

Supplement: Supplementary Figure 5 — Model for a genetically determined, serine-based and phosphorylation-dependent switch that modulates the signaling capacity of FcαRI. The naturally occurring FcαRI-Ser248 is phosphorylated by CK1δ which enhances recruitment of SH3BP5 (Sab), a Btk inhibitor, diminishes recruitment of Lyn kinase, and inhibits Btk-mediated FcαRI receptor signaling (left panel). Dephosphorylation of Ser248 or replacement by the naturally occurring Gly248 genetic variant diminishes Sab recruitment, enhances recruitment of Lyn kinase, and enables activation of receptor signaling (right panel). [file Image5.jpeg]
